# Metabolic regulatory crosstalk between tumor microenvironment and tumor-associated macrophages

**DOI:** 10.7150/thno.51777

**Published:** 2021-01-01

**Authors:** Degao Chen, Xiaomei Zhang, Zhongjun Li, Bo Zhu

**Affiliations:** 1 Institute of Cancer, Xinqiao Hospital, Third Military Medical University, Chongqing 400037, China; 2 Department of Blood Transfusion, Lab of Radiation biology, Xinqiao Hospital, Third Military Medical University, Chongqing 400037, China; 3 Chongqing Key Laboratory of Immunotherapy, Xinqiao Hospital, Third Military Medical University, Chongqing 400037, China

**Keywords:** Tumor-associated macrophage, tumor microenvironment, metabolism, tumor immunotherapy

## Abstract

Macrophages phagocytize pathogens to initiate innate immunity and products from the tumor microenvironment (TME) to mediate tumor immunity. The loss of tumor-associated macrophage (TAM)-mediated immune responses results in immune suppression. To reverse this immune disorder, the regulatory mechanism of TAMs in the TME needs to be clarified. Immune molecules (cytokines and chemokines) from TAMs and the TME have been widely accepted as mutual mediators of signal transduction in the past few decades. Recently, researchers have tried to seek the intrinsic mechanism of TAM phenotypic and functional changes through metabolic connections. Numerous metabolites derived from the TME have been identified that induce the cell-cell crosstalk with TAMs. The bulk tumor cells, immune cells, and stromal cells produce metabolites in the TME that are involved in the metabolic regulation of TAMs. Meanwhile, some products from TAMs regulate the biological functions of the tumor as well. Here, we review the recent reports demonstrating the metabolic regulation between TME and TAMs.

## Introduction

Macrophages that reside within the tumor microenvironment (TME) are known as tumor-associated macrophages (TAMs). As the predominant infiltrated immune cells in the TME 1-3, TAMs have been extensively studied for their pro-tumoral activities, such as tumor initiation, angiogenesis, metastasis, drug-resistance, and antitumor immunosuppression 4. To mirror the Th1/2 immune response, canonical concepts suggest that there are two distinct states of polarized activation in macrophages, namely classical (M1, induced by lipopolysaccharide and IFN-γ) and alternative (M2, induced by IL-4 or IL-13) 5. M1 and M2 macrophages have different transcriptional profiles, such as cytokines, chemokines, metabolic pathways. Studies have indicated that TAMs predominantly have an M2-like phenotype, which manifests as an immunosuppressive state and pro-tumoral progression 6. Hence, targeting M2-like TAMs and depleting them in the TME or reversing the M2-like TAMs into an M1-like phenotype, which directly boosts their cytotoxicity and indirectly stimulates cytotoxic T cells to eliminate tumor cells, is a potential strategy for antitumor immunotherapy 7, 8. Numerous mechanisms underlying the role of TAMs in tumor immunosuppression have been elucidated, and novel therapeutic agents based on these new targets have been subjected to clinical trials in recent years 8, 9. In addition, some recent reports focused on phagocytosis, the primordial function of macrophages, to reactivate antitumor immunity by targeting CD47/SIRPα 10, PD-1/PD-L1 11, and CD24/Siglec-10 12 pathways. All of these strategies have moved TAMs to the forefront of tumor immunotherapy.

In addition to the cytokine and chemokine regulatory mechanisms, metabolic regulation between the TME and TAMs has also been widely studied. All members of the TME rely on nutrients for their survival, maintenance, and proliferation. Meanwhile, the competition and symbiosis between TAMs and other members of the TME form a crosstalk mechanism linked by metabolites. Excessive metabolites uptake or secretion reprograms the phenotype and function of such components and interferes with tumor outcomes. Therefore, clarifying the metabolic regulatory mechanisms would help elucidate the landscape of metabolite connections between the TME and TAMs and promote metabolic immunotherapy targeting TAMs. Some excellent reviews have focused on TAM metabolism and the metabolic crosstalk between tumor cells and TAMs 13-15. However, other than tumor cells, the metabolic regulation of immune and stromal cells in TME also needs to be concerned, especially the interaction with TAMs. Besides, the metabolism pattern of TAMs affects the outcome of cancer. This review discusses the metabolic crosstalk from TME to TAMs based on different cell types and from TAMs to TME by diverse oncobiological functions.

## Metabolic crosstalk: from TME to TAMs

**Figure 1:**
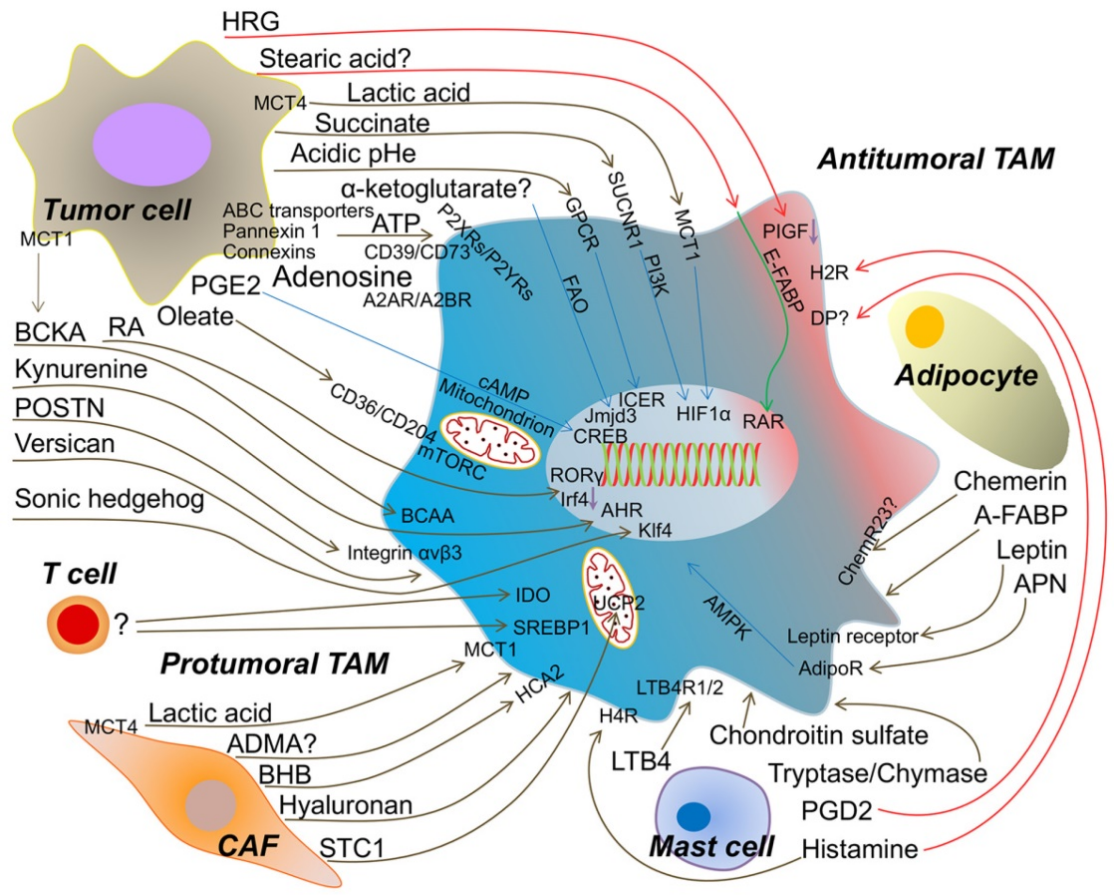
The metabolic crosstalk: from TME to TAMs. Tumor cell-derived lactic acid, succinate, α-ketoglutarate, ATP/Adenosine, PGE2 (prostaglandin E2), oleate, Retinoic acid (RA), BCKA (branched-chain ketoacids), kynurenine, POSTN (periostin), versican, sonic hedgehog and the extracellular acidosis promote the protumoral TAM formation to accelerate tumor progression. Artificially increasing HRG (histidine-rich glycoprotein) or stearic acid expression from tumor cell induce the antitumoral TAM. Metabolites from T cell to TAM are rarely identified, but activated T cell enhances IDO and SREBP1 (sterol regulatory element-binding protein 1) in TAM to facilitate tumor growth. CAF (cancer-associated fibroblast)-produced lactic acid, ADMA (asymmetric dimethyl arginine), BHB (beta-hydroxybutyrate), hyaluronan and STC1 (stanniocalcin-1) assist TAM to be protumoral. Mast cell-released LTB4 (leukotriene B4), chondroitin sulfate and tryptase/chymase guide TAM toward into tumor promoting. However, PGD2 (prostaglandin D2) from mast cell may bind DP (PGD2 receptors) on TAM and skews TAM to be antitumoral. Histamine triggers H4R (histamine H4-receptor) on macrophage into protumoral TAM, but H2R (histamine H2-receptor) into antitumoral TAM. Adipocyte-derived APN (adiponectin), leptin and A-FABP (adipose fatty acid binding protein) induce the protumoral TAM. Chemerin from adipocyte causing the phenotypic change of TAM is tumor context dependent. MCT1/4 (monocarboxylate transporter 1/4); SUCNR1 (succinate receptor1); GPCR (G protein-coupled receptors); FAO (fatty acid oxidation); P2XRs/P2YRs (P2 purinergic receptors); A2AR/A2BR (adenosine receptors); BCAA (branched-chain amino acid); HCA2 (hydroxycarboxylic acid receptor 2); LTB4R1/2 (leukotriene B4 receptor 1/2); AdipoR (adiponectin receptor); ChemR23 (chemokine-like receptor 1, CMKLR1); PIGF (placental growth factor); E-FABP (epidermal fatty acid binding protein); ICER (inducible cyclic AMP (cAMP) early repressor); CREB (cyclic AMP-responsive element binding); AHR (aryl hydrocarbon receptor); RAR (retinoid acid receptor); UCP2 (uncoupling protein 2).

TAMs and other TME members make up the tumor ecosystem, suggesting that there are some interactions between them through cytokines, chemokines, and other factors 16. Most often, all members of the TME consume oxygen and nutrients from the host for their phenotypic and functional performance 17, 18. Thus, metabolites are accumulated in the TME and recycled from cell to cell. In particular, as messengers for cell-cell contact, the metabolites, which are derived from the TME (tumor cells, T cells, mast cells, cancer-associated fibroblasts, adipocytes, except TAMs), are ingested by TAMs to change their phenotype and function. In turn, TAMs promote tumor progression via metabolic reprogramming, which is triggered by the metabolites that are shuttled in the TME. Moreover, blockade of the metabolic pathways in the TME and TAMs is being used for drug discovery and tumor therapy 8, 19. Next, we describe the recent findings regarding metabolic crosstalk between the TME and TAMs (Figure 1).

### Tumor cells

The bulk of cells in the TME are tumor cells. Tumor cells possess the self-serving characteristic of educating the TME to provide pro-tumoral conditions. Numerous tumor cells deprive glucose from the TME for their progression 20. Thereby, other cells, including TAMs, have no alternative but mainly rely on oxidative phosphorylation (OXPHOS) to produce energy for cellular processes 21. However, most tumor cells do not fully transmit glucose into the mitochondrial tricarboxylic acid (TCA) cycle to generate ATP (adenosine triphosphate) efficiently, but instead use glucose in aerobic glycolysis, which is termed the “Warburg Effect” 22. Thus, tumor cells produce a large amount of lactic acid through aerobic glycolysis and release the redundant lactic acid into the extracellular microenvironment via monocarboxylate transporter 4 (MCT4) 23.

Reports indicate that lactic acid-treatment suppresses TNF secretion in human monocytes through glycolysis inhibition 24. Furthermore, lactic acid is reportedly the messenger between tumor cells and TAMs. In addition, tumor cell-derived lactic acid induces the expression of vascular endothelial growth factor (VEGF) and the M2-like polarization of TAMs, which is mediated by hypoxia-inducible factor 1α (HIF1α) 25. Moreover, a recent study demonstrated that lactic acid induces M2-like gene activation in macrophages through histone lactylation 26. Besides, extracellular acidosis is involved in tumor progression via the stimulation of autophagy and immunosuppression 23, which was also found to promote tumor progression by increasing tumor-promoting macrophages in prostate cancer 27. Regarding the underlying mechanism, tumor acidosis is sensed by G protein-coupled receptors (GPCRs) in TAMs. It induces transcriptional repressor ICER (inducible cyclic AMP (cAMP) early repressor) expression to enhance pro-tumoral macrophage polarization, especially in tumors with a high glycolytic rate, such as melanoma 28. It is noteworthy that tumor-derived lactic acid was found to be dispensable for the induction of ICER expression in TAMs in this study 28, which means that other organic acids and hydrogen ions together, with or without lactic acid, contribute to prime TAMs for tumor growth.

Interestingly, the restricted OXPHOS in tumor cells is accompanied by glutamine consumption to fuel the TCA cycle. Glutamine is converted to α-ketoglutarate by glutamate dehydrogenase or aminotransferases 29. One study has reported that intracellular α-ketoglutarate leads to the M2-like activation of macrophages via FAO (fatty acid oxidation) and Jmjd3-dependent epigenetic reprogramming of M2 genes 30. However, more research is needed to elucidate the mechanism of α-ketoglutarate shuttling between tumor cells and TAMs. Succinate, the downstream product of α-ketoglutarate, is an intermediate of the TCA cycle in mitochondria, and its levels are mainly controlled by succinate dehydrogenase (SDH), which is regarded as a tumor suppressor 31. Inherited or somatic mutations and the inhibition of SDH result in tumor formation, which importantly causes the accumulation of succinate in tumor cells 32. Nevertheless, the excessive uptake of glutamine by tumor cells induces glutamine-based anaplerosis, and the γ-aminobutyric acid shunting pathway also increases succinate levels 33. The tumor-derived succinate is taken up through succinate receptor (SUCNR1) and results in the polarization of TAMs into a pro-tumoral form via the SUCNR1/PI3K/HIF1α signaling pathway to enhance tumor metastasis, which can also be mediated by autocrine succinate 34. These studies suggest that serum succinate could be a potential clinical biomarker.

ATP has long been known as the energy molecule. Hypoxia and chemotherapy lead to the accumulation of abundant ATP in the TME 35, which is primarily derived from tumor cells via ATP-binding cassette (ABC) transporters 36, pannexin 1 37, or connexins 38. The extracellular ATP triggers the P2 purinergic receptors (P2XRs and P2YRs) in tumor cells for its growth or inhibition 39. In most cases, ATP is unstable in biological fluids and is rapidly degraded to adenosine, which is abundantly accumulated in extracellular fluids of solid tumors for tumor immunosuppression 40. This biological process is mediated through the ectonucleotidases CD39, which converts ATP into ADP/AMP, and CD73, degrading AMP into adenosine 41. One study reported that adenosine induces TAMs to express CD39 and CD73 for tumor immune escape 42. Moreover, adenosine receptors (A1R, A2AR, A2BR, and A3R) have been found to sense adenosine for tumor growth, survival, metastasis, and immunosuppression 38. Furthermore, reports suggest that CD39/CD73 and adenosine receptors are mainly induced and regulated by HIF1α 43-45. Furthermore, A2AR and A2BR were reportedly to be expressed on macrophages and stimulated by adenosine, inducing M2-like TAM polarization 38, 41. Additionally, A2AR depletion on myeloid cells, especially macrophages, slows melanoma growth and reduces metastasis by relieving T and NK cell suppression 46. Studies have recently revealed that ectonucleotidases, adenosine receptors, and the adenosinergic pathway are potential targets to enhance antitumor immunity 38, 40, which would accelerate drug discovery in this field.

Prostaglandin E2 (PGE2), a prostanoid lipid, is associated with tumor progression 47 and is synthesized by tumor cyclooxygenase to mediate tumor immune escape 48. Reports suggested that PGE2 induces M2 polarization in macrophages through the cyclic AMP-responsive element binding (CREB) pathway 49, 50. Furthermore, PD-L1 expression in TAMs can be regulated by PGE2 levels 51. Recently, one study clarified that PGE2 modulates mitochondrial membrane potential to regulate M2-like gene expression in the nucleus 52. Thereby, PGE2 from tumor cells polarizes TAMs through cAMP and mitochondrial signaling.

Fatty acids are reportedly enriched in the TME and mainly tumor cell-derived 53. A recent paper indicated that long-chain fatty acids, exemplified by oleate, are scavenged by CD36/204, facilitate mitochondrial respiration in TAMs, and polarize immune-suppressive TAMs through mTORC signaling 54. Thus, tumor lipid metabolites orchestrate a TAM phenotype, and this might explain why a lipid droplet-rich TME is associated with more infiltrating TAMs. Nevertheless, researchers revealed that free fatty acids, particularly stearic acid, facilitate the inflammatory functions of CD11c^+^ macrophages via activation of the nuclear retinoic acid receptor and the cytosolic expression of epidermal fatty acid-binding proteins (E-FABP) in obesity models 55. Therefore, whether various categories of fatty acids have different effects on TAM polarization needs more clarification.

Retinoic acid (RA) is a metabolite of vitamin A that has an established role in organ development 56, cancer therapy 57, and immune homeostasis 58.* In vitro*, RA-treated head and neck squamous cell carcinoma cells fail to activate macrophages by decreasing VEGF and IL-8 secretion 59. In addition, the clinical use of available all-trans-retinoic acid (ATRA) abrogates the pro-tumorigenic phenotype of TAMs in prostate cancer by suppressing the activation of NF-κB p50 60 and reduces osteosarcoma metastasis by downregulating MMP12 (matrix metalloproteinase 12) secretion from M2-type TAMs 61. Further, ATRA could prevent osteosarcoma initiation and stemness by inhibiting M2-like TAMs 62. However, retinoic-acid-related orphan receptor (RORC1/RORγ), one of the retinoid nuclear receptors, has been found to prevent myeloid-derived suppressor cells (MDSCs) from undergoing apoptosis, and to promote M2-like TAM differentiation in the TME 63. Moreover, a recent study indicated that tumor cell-derived RA promotes TAMs and suppresses immunostimulatory dendritic cell differentiation from monocytes 64. Combined with the RA mechanism in treating acute promyelocytic leukemia, the concept of differentiation therapy, and hence, RA from tumor cells, seems more like an immunosuppressant to mediate TAM formation in the TME.

Nevertheless, the effects of other tumor cell-derived metabolites on TAMs have been investigated. Branched-chain ketoacids, which are excreted from glioblastoma cells via monocarboxylate transporter 1 (MCT1), are taken up by TAMs and re-purposed to form branched-chain amino acids, which reduces phagocytosis by TAMs and mediates tumor immunosuppression 65. Moreover, a recent study revealed that glioblastoma cell-derived kynurenine activates AHR (aryl hydrocarbon receptor) in TAMs to modulate TAM recruitment and T cell dysfunction 66. With respect to glioblastoma, researchers found that periostin (POSTN) from glioma stem cells (GSCs) recruits monocyte-derived macrophages from the peripheral blood through integrin αvβ3 to induce an M2 phenotype, and POSTN knockdown in GSCs inhibits tumor growth 67. One report revealed that bladder tumor cell-derived versican drives lung metastasis in a TAM dependent manner 68; however, the mechanism underlying CCL2 expression in TAMs via versican warrants additional investigation. Another recent study reported that tumor cell-derived sonic hedgehog triggers hedgehog signaling in TAMs for M2 polarization and impedes CD8^+^ T cell recruitment by hindering CXCL9 and CXCL10 production by TAMs, which is mediated by Kruppel-like factor 4 (Klf4) 69. Meanwhile, some tumor metabolites can re-educate TAMs to induce antitumor activity. HRG (histidine-rich glycoprotein) levels are decreased in the TME, and researchers have increased tumor levels by using a genetic gain-of-function strategy to find that HRG derived from these tumor cells inhibits tumor growth and metastasis by inducing macrophage polarization and vessel normalization through the downregulation of PIGF (placental growth factor), suggesting that HRG contributes to the skewing of TAMs to an antitumor polarization phenotype 70.

### T cells

T cell-based immune-checkpoint inhibitors have resulted in tremendous achievements 71. Given that T cells are the cytotoxic cells of the antitumor immune response, their persistent survival, and the production of toxicity factors are critical to eliminate tumor cells and surveil tumor initiation and recurrence. All of these functions are based on metabolic support 72. The naïve T cells mainly use the OXPHOS pathway to produce ATP 73. When activated by immunological signals, the metabolic model switches to be glycolysis-dependent 74. In the TME, glucose, oxygen, and other essential metabolites are deprived by tumor cells. T cells must thus adapt to the hypoxic and nutrient-deficient conditions, which becomes tumor immunosuppressive. Thus, the TME reprograms T cell metabolism into weak glycolysis and OXPHOS, accompanying the decrease in IFN-γ and perforin/granzyme B secretion 72. However, the impact of T cell-derived metabolites on TAMs has not received much research attention with respect to the TME. One report indicated that tumor-infiltrating CD69^+^-activated T cells increase IDO (indoleamine 2,3-dioxygenase) expression in TAMs, but the metabolic mediator has not been identified 75. Another group revealed that CD8 T cell-derived IFN-γ could block sterol regulatory element-binding protein 1 (SREBP1)-mediated fatty acid metabolism in TAMs 76. In summary, cytokine/chemokine crosstalk is common between T cells and TAMs, but metabolites are also important.

### Mast cells

Mast cells are derived from bone marrow precursors and reside in peripheral tissues after maturation; further, they play the role of sentinels to recruit an immune army during infection, especially in allergic responses 77. To date, the beneficial effects of mast cells on both the tumor and the host have been reported 78, 79. A recent report found that IL-33-mediated mast cell activation in gastric cancer enhances TAM infiltration and tumorigenesis through CSF2, CCL3, and IL-6 secretion 80. Other than cytokines and chemokines, mast cells also secrete distinct biogenic amines, eicosanoids, and proteoglycans when they are activated, which are either pro- or anti-tumoral. Theoharis C. Theoharides proposed M1- and M2-type mast cells according to their different effects on the tumor 78. Moreover, some of these molecules have been studied on TAMs.

Histamine, one of the biogenic amines from mast cells, combined with IL-2, increases Th1 immune responses in patients with stage IV melanoma 81. Later clinical data suggested that histamine with IL-2 does not change the number of intratumoral macrophages 82. With respect to the mechanism, histamine can interact with the H2-receptor on macrophages to inhibit ROS production, which relieves NK and T cell inhibition to favor tumor inhibition 83. Further, histamine deficiency causes a high incidence of colon and skin carcinogenesis via abnormal myeloid differentiation 84. Moreover, macrophages also express other histamine receptors, including H1/4-receptor, and the H4-receptor is required to induce histamine-mediated chemotaxis and phagocytic activity 85. Further, H4-receptor knockout mice show suppressed mammary tumor growth and metastasis through a reduction in CD4 T and Treg cells and an increase in NK cells in tumor-draining lymph nodes; besides, stimulation of the H4-receptor increases Treg numbers, enhances IL-10 expression, and decreases IFN-γ 86. Moreover, the same group recently found that histamine has an antitumor effect, with increased cytotoxic lymphocyte infiltration, in H4-receptor knockout mice 87. Thus, the specific histamine receptor that induces the antitumor immune response is still unknown. Histamine is generated by histidine decarboxylase (HDC), and HDC-knockout mice fail to form M1-like macrophages 88. To date, although no systematic study has revealed the phenotypic and functional effect of histamine on TAMs, we speculate that histamine could polarize M1-like TAMs, based on these data combined with results of a previous report 89.

Activated mast cells can produce three eicosanoids, prostaglandin D2 (PGD2), leukotriene B4 (LTB4), and LTC4 90. These three eicosanoids are produced *de novo* from arachidonic acid derived from nuclear membrane phospholipids via cytosolic phospholipase A2. Prostaglandin H2 (PGH2) is generated from arachidonic acid by prostaglandin H synthase 1/2 (PGHS1/2, also called cyclooxygenase 1/2) and then is converted to PGD2 by hematopoietic prostaglandin D2 synthase (H-PGDS, in leukocytes) or lipocalin prostaglandin D2 synthase (L-PGDS, in the central nervous system). PGD2 has been found to induce leukocyte chemotaxis via PGD2 receptors (DP) in Th2 cells 91. Enhancing the PGD2/DP pathway results in a suppressive effect on tumor growth by modulating tumor hyperpermeability and angiogenesis 92. Moreover, decreasing PGD2 through H-PGDS deficiency increases tumor growth, and mast cell-derived PGD2 reduces tumor expansion 93. These findings suggest that the PGD2/DP pathway favors the inhibition of tumor progression. Although many studies mentioned PGD2 in the TME 94, DP expression and the regulation of PGD2 biosynthesis in TAMs are yet to be identified. Mast cell-derived leukotrienes are potent pro-inflammatory lipid mediators implicated in cancers, which require 5-lipoxygenase (5-LOX) for their production. Generally, 5-LOX plays a pro-carcinogenic role 95, whereas its repression in TAMs also promotes tumor progression 96. Thus, the effect of the 5-LOX pathway on tumors is diverse and might depend on the metabolites from 5-LOX. LTB4, one of several leukotrienes derived from mast cells, binds to two GPCR LTB4Rs (LTB4R1 with high affinity and LTB4R2 with low affinity 47) and favors murine melanoma growth 97. Elevated TAMs can be attracted to the TME inducing immunosuppression via LTB4 98. The addition of LTC4 has no remarkably increasing effect on the tumoricidal function of TAMs 99. Leukotrienes and the expression of their receptors, especially LTB4, are increased in some human cancers, including colon, prostate, and pancreatic cancer 47. Except with respect to chemotaxis functions, other mechanisms of leukotriene-mediated TAM polarization are comparatively less well known.

For proteoglycans, mast cell-derived heparin has been reported to increase angiogenesis 100. However, the interaction between heparin and TAMs has not been elucidated. Chondroitin sulfate, another proteoglycan from mast cells, is associated with decreased T cells and increases in TAM infiltration in the TME 68, 101. However, the use of chondroitinase gene therapy to digest chondroitin sulfate increases CD206 (a marker of M2-like macrophages) expression after spinal cord contusion injury 102. These data suggest that chondroitin sulfate deficiency might favor wound healing. Thus, further clarification of the mechanism underlying the effect of mast cell-derived proteoglycans on TAMs might explain the different functional changes. Mast cells are typically characterized by protease storage and secretion, including tryptase and chymase 103. The expression of these two enzymes correlates with tumor progression 104. These enzymes' primary function is to degrade the extracellular matrix, which might indirectly mediate the immune response in the TME. However, fewer studies have mentioned the impact on TAMs.

In general, mast cells were known to produce chemotactic materials. These chemoattractants induce immune responses in the TME, and especially, TAMs have been overlooked. Increasing research findings indicate that the number of immune cells in the TME is not the crucial factor with respect to tumor elimination, and increasing the tumoricidal immune cells by regulating these metabolites might be the future task, for instance, by reversing the M2-like phenotype of TAMs to generate antitumor M1-like macrophages with these mast cell-derived metabolites.

### Cancer-associated fibroblasts

The tumor stroma constructs the framework of a solid tumor. Cancer-associated fibroblasts (CAFs), which can be derived from endothelial cells, pericytes, adipocytes, and mesenchymal stem cells 105, are the dominant component of the tumor stroma and have been suggested to boost tumor progression and metastasis 106, 107. A variety of makers have been used to identify CAFs, such as α-SMA (smooth muscle actin), FSP1 (fibroblast specific protein 1), NG2 (neuron-glial antigen-2), and CD90, among others 105. To date, CAFs cannot be identified with a single marker, even in the same tumor. In line with this, CD10^+^GPR77^+^ CAFs have been found to promote tumor formation and chemoresistance by sustaining tumor stemness 108. Studies have indicated that fibroblast activation protein and microRNAs can reprogram normal fibroblasts into CAFs 109. Moreover, CAFs and TAMs are often used together to assess tumor progression 110.

In recent years, some discoveries in CAF metabolism have been made. Michael P. Lisanti's group has presented many studies on metabolism in CAFs, and they termed this new paradigm “The Reverse Warburg Effect” 111, which is characterized by increased lactate and pyruvate secretion from CAFs to fuel tumor cells 112. As in the tumor cells, they found that lactate is released from CAFs via MCT4 113, 114. Even though no study has determined whether lactate from CAFs differentially affects TAMs than that from tumor cells, we believe that CAF-derived lactate polarizes M2-like TAMs, as previously mentioned herein. Moreover, they found that the loss of caveolin-1 (Cav-1) in CAFs is associated with tumor progression 115. Asymmetric dimethyl arginine (ADMA) and beta-hydroxybutyrate (BHB), which are markers of oxidative stress and mitochondrial dysfunction, are profoundly upregulated in Cav-1-knockout CAFs 116. Interestingly, endogenous ADMA has been demonstrated to inhibit nitric oxide synthase activity, probably inducing immune-suppressive TAMs in the TME 117; however, more evidence is needed to indicate if ADMA can be taken up into TAMs. Furthermore, BHB, a ketone body produced by β-oxidation, also induces anti-inflammatory macrophages through HCA2 (hydroxycarboxylic acid receptor 2) 118. Therefore, ADMA and BHB from CAFs might be conducive to TAM polarization, but the two metabolites' primary source needs to be fully elucidated.

Additionally, other CAF-specific metabolites have been found to affect TAMs. It has been found a high level of hyaluronan in the TME predicts poor prognosis for tumor patients 119. Nobutaka Kobayashi and co-workers found that TAMs preferentially traffic to hyaluronan-enriched areas, and genetic disruption of the hyaluronan synthase 2 (*Has2*) gene in fibroblasts impairs TAM recruitment and reduces tumor angiogenesis and lymphangiogenesis, which indicates that CAF secretion of hyaluronan results in TAM pro-tumoral activities 120. Besides, stanniocalcin-1 (STC1), which is secreted by CAFs, is recognized as a mediator of colorectal cancer growth and metastasis 121. The previous studies suggested that STC1 is an anti-inflammatory protein, especially for macrophages, which is mediated by inducing mitochondrial UCP2 (uncoupling protein 2) to suppress superoxide generation 122. Thus, STC1 might inhibit the antitumor chemokine expression in TAMs to impede antitumor T cell infiltration. In summary, very few studies have been performed to assess CAF metabolites' effect on TAMs, except for some specific metabolites.

### Adipocytes

Besides CAFs, as another type of stromal cell, adipocytes, also called cancer-associated adipocytes (CAAs), are through to promote tumor growth and metastasis by secreting inflammatory factors and metabolites in the TME 123, especially in breast cancer 124. CAAs have been reported to increase tumor growth and vascularization through TAMs 125. Meanwhile, the impact of metabolites from CAAs on TAMs has been studied. For example, adiponectin (APN), one such adipokine, is produced by adipocytes and has antitumor activities 126 and pro-tumoral activities 127. In response to APN, macrophages are mainly characterized by their anti‐inflammatory effect 128 and M2-like phenotype, specifically, negatively regulating the growth of macrophage progenitors 129, inhibiting the production of CXCR3 ligands to reduce T cell migration 130, and decreasing the production of pro-inflammatory cytokines by suppressing IκB, JNK, p38, and STAT3 phosphorylation 131, 132. Likewise, full-length APN was reported to induce M2 macrophage polarization via AMPK and shift macrophage metabolism to OXPHOS 133. Moreover, in the TME, APN deficiency induces TAM polarization to an M1-like phenotype to favor an antitumor immune response for tumor inhibition 134. In mechanistic terms, APN might trigger downstream signaling of adiponectin receptors (AdipoRs) to polarize M2-like TAMs, such as AdipoR1 135, 136, AdipoR2 136, 137, C1qRp 129, and calreticulin receptor 138, among others. AdipoR1/2 probably mediates this on TAMs, which is suggested by the observation that AdipoR1/2 expression on dendritic cells mediates T cell anergy and tolerance in breast cancer 139.

Leptin, another adipokine, was also found to shape the TME and contribute to tumor development 140. In macrophages, leptin induces some inflammatory mediators' production, including TNF-α, IL-6, and leukotriene B4 141. Moreover, leptin modulates cellular lipid metabolism and storage by enhancing ADRP (adipose differentiation-related protein) accumulation within macrophages via the PI3K/mTOR pathway 141. Although the phenotypic changes in TAMs mediated by leptin have not been revealed, leptin-mediated macrophage chemotaxis and activation were demonstrated through the leptin receptor, which was found to require JAK/STAT, MAPK, and PI3K pathways 142. This indicates that leptin might mediate the link between CAAs and M2-like macrophages in high-fat diet-induced obesity-associated tumor metastasis 143.

Chemerin, a novel adipokine, regulates adipogenesis and adipocyte metabolism in an autocrine and paracrine manner 144 and was found to be a potent chemoattractant for macrophages that functions in a ChemR23-dependent manner 145. ChemR23, also known as chemokine-like receptor 1 (CMKLR1), is downregulated in mouse M1-like macrophages but upregulated in mouse M2-like macrophages 146. However, subsequent research suggested that human M1-like macrophages express ChemR23 but human M2-like macrophages do not 147. In DSS-induced colitis, chemerin aggravates mucosal damage and increases pro-inflammatory cytokines by suppressing M2-like macrophage polarization 148. This suggests that the mechanism of chemerin-induced inflammation is intricate. In the TME, chemerin has been reported to inhibit tumor growth by increasing NK cell infiltration and reducing MDSC accumulation 149. Furthermore, chemerin-deficient (Rarres2^-/-^) mice show significantly increased TAM numbers in the hepatocellular carcinoma TME 150. Moreover, tumor cells favor CMKLR1 expression in macrophages, and chemerin enhances pro-inflammatory gene expression in TAMs 151. This reveals that chemerin might skew TAMs to have an antitumor effect. However, chemerin is downregulated in most tumor tissues, including melanoma, acute myeloid leukemia, breast cancer, and adrenocortical carcinoma 152, indicating that chemerin downregulation can induce tumor immune escape. Thus, the re-introduction of chemerin into the TME might represent a therapeutic strategy 153. Meanwhile, chemerin is elevated in colorectal cancer, gastric cancer, glioblastoma, hepatocellular carcinoma, and others 152. Therefore, the effect of treating tumors with chemerin could be tumor context-dependent, and further study is warranted. In addition, recent research indicated that circulating adipose fatty acid binding protein (A-FABP) is mainly derived from CAAs, and elevated circulating A-FABP is associated with obesity-associated tumor development 154. However, the impact of A-FABP on TAMs is still elusive, since CD11b^+^F4/80^+^MHCII^-^Ly6C^-^ TAMs produce A-FABP as well 155. Together, the metabolite-mediated interaction between CAAs and TAMs is complicated, and further studies are urgently needed.

Although cellular metabolism in the TME is different from that in normal host cells, all cells share the same metabolic pathways 156. It is difficult to find some specific metabolites from one component of the TME, but it is feasible to analyze the quantity of various metabolites based on corresponding key metabolic enzymes' expression. In general, the quantity of metabolites in early tumor patients is relatively small. Thereby, the diagnostic validity of metabolic tumor markers is limited to advanced and metabolically hyperactive tumors. TAMs are surrounded by the bulk of tumor cells and other components in the TME in either early- or late-stage tumors. Even slight changes in TME metabolic secretions could shape the immune phenotype of TAMs. However, it is a technical challenge to detect changes in local TME metabolites directly.

## Metabolic crosstalk: from TAMs to TME

**Figure 2:**
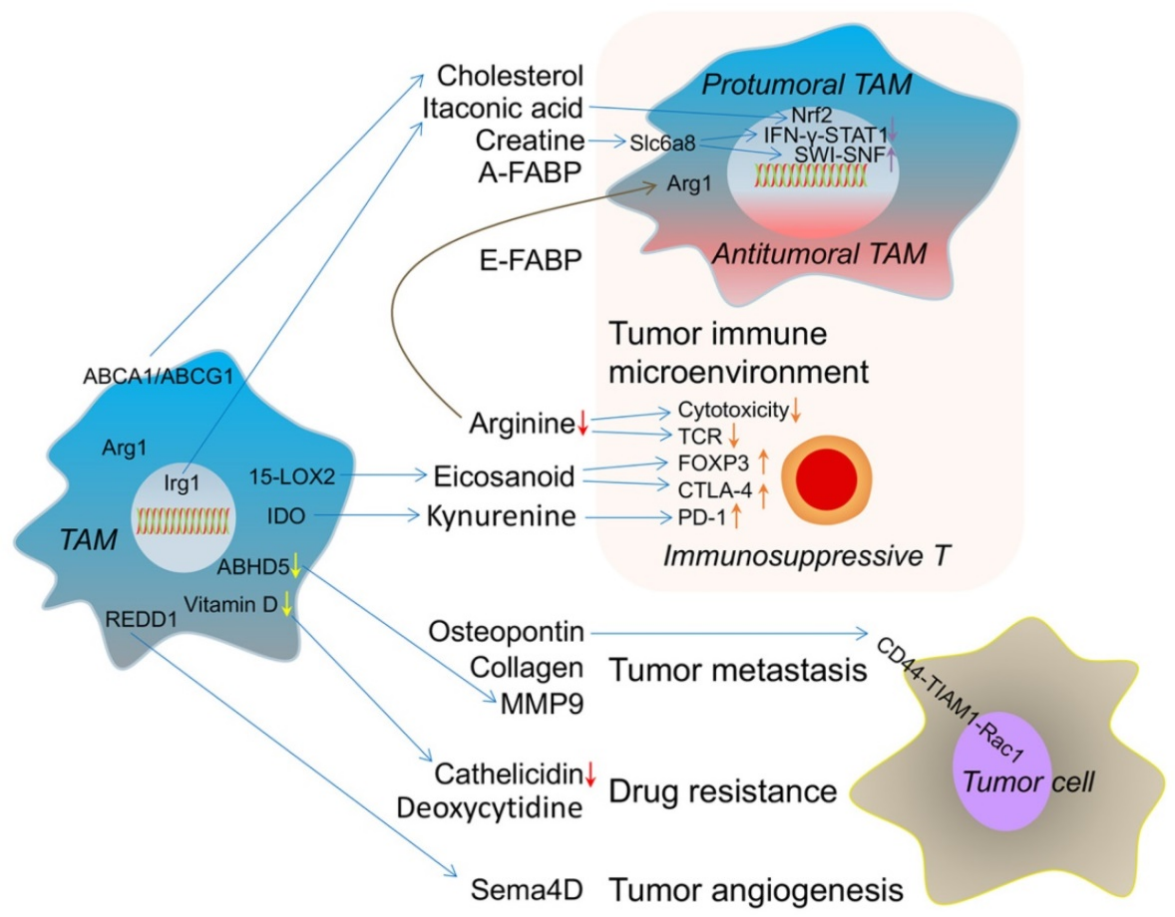
The metabolic crosstalk: from TAMs to TME. TAM-derived cholesterol, itaconic acid, creatine and A-FABP switch its own phenotype into protumoral TAM. E-FABP (epidermal fatty acid binding protein) from TAM induces the antitumoral TAM formation. TAM-caused arginine consumption, eicosanoid and kynurenine accumulation result in immunosuppressive T cell. These effects reflect the immune microenvironment changes induced by metabolites from TAM. TAM-produced osteopontin, collagen and MMP9 (matrix metalloproteinase 9) promote tumor metastasis. Lower vitamin D-caused decreased cathelicidin in TAM and TAM-released deoxycytidine induce drug resistance. REDD1(regulated in development and DNA damage response 1)-dependent Sema4D (semaphorin 4D) secretion from TAM accelerates tumor angiogenesis. ABHD5 (abhydrolase domain-containing 5); 15-LOX2 (15-lipoxygenase-2); Irg1 (immune-responsive gene 1).

Metabolites from the TME affect the polarization of TAMs to induce tumor progression and inhibition. In turn, TAMs regulate the TME as well, such as via known cytokines and vesicles. As mentioned previously herein, tumor tissues are hypoxic, and tumor cells mainly use aerobic glycolysis to generate energy. TAMs have a direct impact on tumor cell metabolism. A recent study reported that TAMs make non-small cell lung cancer (NSCLC) cells more glycolytic through TNF-α secretion and promote enhanced hypoxia through AMP-activated protein kinase and PGC-1α activation. Moreover, the depletion of TAMs increases T cell infiltration and PD-L1 expression in tumor cells, which is beneficial for the PD-L1 blockade in NSCLC 157. Another recent study suggested that enhanced aerobic glycolysis and decreased apoptosis in breast cancer cells are mediated by TAM-derived extracellular vesicle-packaged HIF-1α-stabilizing long noncoding RNA, which is upregulated by lactate in the TME 158. An additional recent report focused on clear cell renal cell carcinoma, which highly relies on glutamine, and found that glutamine-deprived TAMs secrete IL-23 to decrease survival and enhance the immunosuppressive function of Tregs 159. In addition to the altered tumor cell metabolism mediated by TAM-derived cytokines and vesicles, metabolites from TAMs extensively regulate the tumor immune microenvironment, metastasis, angiogenesis, and drug-resistance (Figure 2).

First, some secreted molecules from TAMs can shape their immune phenotype. Reducing circulating cholesterol levels has been considered a useful strategy to treat breast cancer 160. One study recently suggested that membrane cholesterol efflux in TAMs is increased by ovarian cancer cell-derived hyaluronic acid. On the one hand, these TAM-derived cholesterols promote tumor growth, whereas, on the other hand, high cholesterol efflux disrupts lipid rafts in TAMs and results in TAM reprogramming toward a tumor-promoting phenotype 161. The cholesterol efflux in TAMs has a positive correlation with tumor burden. The genetic deletion of ABCA1 and/or ABCG1 revert the tumor-promoting functions of TAMs and reduces tumor growth. Activated macrophages could produce itaconic acid 162, which is regulated by immune-responsive gene 1 (Irg1) and an essential regulator of macrophage metabolism and inflammation 163. Interestingly, itaconic acid acts as a metabolic brake in activated macrophages to avoid hyperinflammatory reactions. In mechanistic terms, itaconic acid inhibits succinate dehydrogenase to reprogram immune responses in macrophages 164 and activates the anti-inflammatory transcription factor Nrf2 via KEAP1 alkylation 165. Importantly, itaconic acid, a key anti-inflammatory metabolite in macrophages, is the most highly upregulated metabolite of TAMs in a peritoneal tumor model and mediates tumor growth. Correspondingly, the genetic downregulation of Irg1 remarkably reduces peritoneal tumors 166. FABPs are known as the carrier of free fatty acids that regulate lipid metabolism and inflammation in the host cells. The tissue distributions and ligand-binding specificities of a series of FABPs have been elucidated 167. In tumors, one report found that F4/80^+^CD11b^+^MHCII^+^CD11c^+^ TAMs highly express E-FABP, which promotes the recruitment of T and NK cells to mediate antitumor effects through IFN-β production 168. However, the same group later found that F4/80^+^CD11b^+^MHCII^-^CD11c^-^ TAMs express adipocyte/macrophage FABP (A-FABP) and promote tumor progression through NF-κB-miR-29b-regulated IL-6 production 155. Therefore, TAMs expressing FABPs represent a double-edged sword for tumor immune surveillance. Moreover, the marker of M2-like TAMs is arginase 1 (Arg1), which depletes arginine to form urea and ornithine^169^. Another result of L-arginine metabolism is the generation of creatine. In addition to ATP, earlier reports indicated that creatine, another direct energy molecule, is likely an antitumor molecule 170. However, a recent study suggested that creatine uptake via Slc6a8 skews the alternative macrophage polarization by suppressing IFN-γ-STAT1 signaling and promoting ATP-dependent SWI-SNF-mediated chromatin remodeling 171. Creatine is highly needed in muscle, liver, and brain tissues 172. Thus, it is assumed that the TME, especially tumors initiated from these organs, maintains high creatine amounts. However, the crosstalk between creatine in the TME and TAMs is not understood. Meanwhile, arginine-generated ornithine is the precursor of polyamines and proline, TAM-derived ornithine might be beneficial for tumor cells and their proliferation 173. Additionally, T cell immune responses are affected by TAM metabolites 174. Early research reported that arginine consumption via Arg1 suppresses T cell receptor expression and T cell cytotoxicity 175, 176. Later, a study found that arginine is critical for T cell survival and antitumor T cell immunity 177. This is one of the metabolic reasons why increased TAM accumulation in tumor tissues hinders the antitumor T cell immune response. Furthermore, the 15-lipoxygenase-2 (15-LOX2) pathway enhances eicosanoid production in renal cell carcinoma TAMs, which increases the expression of FOXP3 and CTLA-4 in T lymphocytes to mediate immunosuppression 178. Tumor-repopulating cell-derived kynurenine, the downstream metabolite of tryptophan via IDO catalysis, upregulates PD-1 expression in CD8 T cells by activating the transcriptional factor AhR 179. This study indicated that metabolites from IDO signaling modulate T cell immunosuppression. It should be noted that IDO is also overexpressed in TAMs 180. In addition, TAM-derived kynurenine might similarly induce immunosuppressive T cells.

Metabolites from TAMs contribute to tumor invasion and metastasis. TAM-secreted microvesicle-packaged microRNAs have been found to promote breast cancer cell invasion 181. Osteopontin from TAMs induces bladder cancer metastasis through the osteopontin-CD44-TIAM1 (T cell lymphoma invasion and metastasis 1)-Rac1 pathway 182. The blockade of this pathway disrupts the initial steps in bladder cancer metastasis. Furthermore, monocyte-derived TAMs upregulate collagenous extracellular matrix, such as collagen types I, VI, and XIV, for tumor invasion 183. MMP9 has been involved in tumor lung-specific metastasis 184. Recently, researchers found that ABHD5 (abhydrolase domain-containing 5) deficiency in TAMs induces the NF-κB p65-dependent production of MMP, which indicates that low-level triglyceride hydrolysis-mediated MMP is one of the mediators of tumor metastasis from TAMs 185.

Moreover, TAMs influence tumor angiogenesis. An earlier study found that high TAM numbers are associated with human breast tumor angiogenesis 186. In terms of the mechanism, cytokines and chemokines are the predominant pro-angiogenic factors produced by TAMs 187. Besides, MMPs 188 and cathepsins 189 from TAMs have been suggested to support tumor angiogenesis through extracellular matrix degradation. Semaphorin 4D, a ligand of plexin B1 on endothelial cells, is mainly derived from TAMs in the TME and promotes tumor angiogenesis and vessel maturation 190. Subsequent research also found that TAMs enhance the abnormal vessel formation that occurs through REDD1 (regulated in development and DNA damage response 1)-mediated mTOR inhibition, and REDD1-deficient TAMs deprive glucose from endothelial cells, with consequent formation of an organized tumor vasculature to prevent metastasis 191. However, the metabolic derivatives of REDD1-deficient TAMs are still unknown.

In principle, macrophages can kill tumor cells via antibody-dependent cellular cytotoxicity (ADCC). For example, the M1-like macrophages target proliferating high-grade B cell lymphoma cells by releasing cathelicidin in a vitamin D-dependent manner. Instead, tumor-educated M2-like TAMs downregulate vitamin D metabolism and produce less cathelicidin resulting in failed cytotoxicity 192. This phenomenon suggests that reduced cathelicidin from TAMs mediates ADCC resistance. Another recent report found that TAMs release deoxycytidine, based on liquid chromatography coupled tandem mass spectrometry metabolomics, which induces gemcitabine resistance by competitively inhibiting gemcitabine uptake in pancreatic ductal adenocarcinoma 193. Using the colony-stimulating factor receptor 1 (CSFR1) inhibitor AZD7507, depleting TAMs prolongs survival in an autochthonous pancreatic cancer model. In summary, metabolites from TAMs affect multiple biological functions of the TME, especially remodeling the tumor immune microenvironment. With the clinical application of immunotherapy, an evaluation of metabolic flux in TAMs might guide tumor immunotherapy and elucidate the mechanism underlying drug-resistance and tumor immunotherapy progression.

## Conclusions and Perspectives

Outstanding achievements have been made with respect to tumor immunotherapy; however, we still cannot completely overcome tumors. Chimeric antigen receptor (CAR)-T therapy effectively saves patients from refractory B cell malignancies, especially acute lymphoblastic leukemia (ALL), but the macrophage-involved cytokine-release syndrome (CRS) and neurotoxicity after CAR-T injection are associated with life-threatening consequences 194, 195. Some cytokines from macrophages have been identified in CAR-T-mediated CRS and neurotoxicity, such as IL-1 and IL-6. However, no metabolites have been clarified in this field. Meanwhile, even after receiving immune-checkpoint inhibitors, a so-called hyperprogressive phenomenon is observed in the clinic 196. The mechanism of hyperprogression in immunotherapy has puzzled clinical immunologists. We might consider that the phenotype of TAMs becomes tumoricidal after immunotherapy, but little is known about the metabolic changes in these cells. An analysis of unique shuttling metabolites between TAMs and the TME might help us understand hyperprogression in immunotherapy.

Even though the M1/2 classification is useful to analyze macrophages *in vitro*, this oversimplification cannot convey the heterogeneity of TAMs *in vivo*. Recent studies have indicated that TAM subsets cannot be explained by the M1/2-associated genes 197-199. Using single-cell RNA sequencing and/or mass cytometry, the immune landscape of the TME has been revealed in human clear cell renal cell carcinoma 200, breast cancer 201, hepatocellular carcinoma 199, brain cancer 202, and colon cancer 203. Thus, combined with metabolomics, the subtype classification of TAMs will more abundantly reflect the heterogeneity of TAMs in the TME, and novel metabolic targets might be discovered.

## Abbreviations

TME: tumor microenvironment
TAM: tumor-associated macrophage
IFN-γ: interforn-γ
SIRPα: signal regulatory proteinα
OXPHOS: oxidative phosphorylation
TCA: tricarboxylic acid
ATP: adenosine triphosphate
MCT1: monocarboxylate transporter 1
MCT4: monocarboxylate transporter 4
TNF: tumor necrosis factor
VEGF: vascular endothelial growth factor
HIF1α: hypoxia-inducible factor 1α
GPCRs: G protein-coupled receptors
ICER: inducible cyclic AMP (cAMP) early repressor
FAO: fatty acid oxidation
SDH: succinate dehydrogenase
SUCNR1: succinate receptor
P2XRs and P2YRs: P2 purinergic receptors
PGE2: prostaglandin E2
CREB: cyclic AMP-responsive element binding
mTOR: mammalian target of rapamycin
RA: retinoic acid
ATRA: all trans-retinoic acid
MMP12: matrix metalloproteinase 12
RORC1/RORγ: retinoic-acid-related orphan receptor
MDSCs: myeloid-derived suppressor cells
AHR: aryl hydrocarbon receptor
POSTN: periostin
GSCs: glioma stem cells
Klf4: Kruppel-like factor 4
HRG: histidine-rich glycoprotein
PIGF: placental growth factor
IDO: indoleamine 2,3-dioxygenase
SREBP1: sterol regulatory element-binding protein 1
HDC: histidine decarboxylase
PGD2: prostaglandin D2
LTB4: leukotriene B4
LTC4: leukotriene C4
PGH2: prostaglandin H2
H-PGDS: hematopoietic prostaglandin D2 synthase
L-PGDS: lipocalin prostaglandin D2 synthase
5-LOX: 5-lipoxygenase
CAFs: cancer-associated fibroblasts
α-SMA: smooth muscle actin
FSP1: fibroblast specific protein 1
NG2: neuron-glial antigen-2
Cav-1: caveolin-1
ADMA: asymmetric dimethyl arginine
BHB: beta-hydroxybutyrate
HCA2: hydroxycarboxylic acid receptor 2
STC1: stanniocalcin-1
UCP2: uncoupling protein 2
CAAs: cancer-associated adipocytes
APN: adiponectin
AdipoRs: adiponectin receptors
ADRP: adipose differentiation-related protein
CMKLR1: chemokine-like receptor 1
A-FABP: adipose fatty acid binding protein
NSCLC: non-small cell lung cancer
Arg1: arginase 1
TIAM1: T cell lymphoma invasion and metastasis 1
ABHD5: abhydrolase domain-containing 5
REDD1: regulated in development and DNA damage response 1
ADCC: antibody-dependent cellular cytotoxicity
CSFR1: colony stimulating factor receptor 1
CAR: chimeric antigen receptor
ALL: acute lymphoblastic leukemia
CRS: cytokine-release syndrome
